# Methylation of *HOXA9* and *ISL1* Predicts Patient Outcome in High-Grade Non-Invasive Bladder Cancer

**DOI:** 10.1371/journal.pone.0137003

**Published:** 2015-09-02

**Authors:** Mark O. Kitchen, Richard T. Bryan, Kim E. Haworth, Richard D. Emes, Christopher Luscombe, Lyndon Gommersall, K. K. Cheng, Maurice P. Zeegers, Nicholas D. James, Adam J. Devall, Anthony A. Fryer, William E. Farrell

**Affiliations:** 1 Institute for Science and Technology in Medicine, Keele University, Stoke-on-Trent, United Kingdom; 2 Urology Department, University Hospitals of North Midlands NHS Trust, Stoke-on-Trent, United Kingdom; 3 School of Cancer Sciences, University of Birmingham, Birmingham, United Kingdom; 4 Advanced Data Analysis Centre, University of Nottingham, Nottingham, United Kingdom; 5 Department of Complex Genetics, NUTRIM School for Nutrition, Toxicology and Metabolism, Maastricht University Medical Centre, Maastricht, The Netherlands; 6 Cancer Research Unit, University of Warwick, Coventry, United Kingdom; Thomas Jefferson University, UNITED STATES

## Abstract

**Introduction:**

Inappropriate DNA methylation is frequently associated with human tumour development, and in specific cases, is associated with clinical outcomes. Previous reports of DNA methylation in low/intermediate grade non-muscle invasive bladder cancer (NMIBC) have suggested that specific patterns of DNA methylation may have a role as diagnostic or prognostic biomarkers. In view of the aggressive and clinically unpredictable nature of high-grade (HG) NMIBC, and the current shortage of the preferred treatment option (Bacillus:Calmette-Guerin), novel methylation analyses may similarly reveal biomarkers of disease outcome that could risk-stratify patients and guide clinical management at initial diagnosis.

**Methods:**

Promoter-associated CpG island methylation was determined in primary tumour tissue of 36 initial presentation high-grade NMIBCs, 12 low/intermediate-grade NMIBCs and 3 normal bladder controls. The genes *HOXA9*, *ISL1*, *NKX6-2*, *SPAG6*, *ZIC1* and *ZNF154* were selected for investigation on the basis of previous reports and/or prognostic utility in low/intermediate-grade NMIBC. Methylation was determined by Pyrosequencing of sodium-bisulphite converted DNA, and then correlated with gene expression using RT-qPCR. Methylation was additionally correlated with tumour behaviour, including tumour recurrence and progression to muscle invasive bladder cancer or metastases.

**Results:**

The *ISL1* genes’ promoter-associated island was more frequently methylated in recurrent and progressive high-grade tumours than their non-recurrent counterparts (60.0% *vs*. 18.2%, *p* = 0.008). *ISL1* and *HOXA9* showed significantly higher mean methylation in recurrent and progressive tumours compared to non-recurrent tumours (43.3% *vs*. 20.9%, *p* = 0.016 and 34.5% *vs* 17.6%, *p* = 0.017, respectively). Concurrent *ISL1/HOXA9* methylation in HG-NMIBC reliably predicted tumour recurrence and progression within one year (Positive Predictive Value 91.7%), and was associated with disease-specific mortality (DSM).

**Conclusions:**

In this study we report methylation differences and similarities between clinical sub-types of high-grade NMIBC. We report the potential ability of methylation biomarkers, at initial diagnosis, to predict tumour recurrence and progression within one year of diagnosis. We found that specific biomarkers reliably predict disease outcome and therefore may help guide patient treatment despite the unpredictable clinical course and heterogeneity of high-grade NMIBC. Further investigation is required, including validation in a larger patient cohort, to confirm the clinical utility of methylation biomarkers in high-grade NMIBC.

## Introduction

High-grade non-muscle invasive bladder cancer (HG-NMIBC) is a clinically important sub-type of bladder transitional cell carcinoma (TCC), accounting for 10–15% of all TCC at presentation[1].The unpredictable nature of HG-NMIBC with regard to recurrence and progression to invasive or metastatic disease, presents many challenges for successful management. With no robust methods for predicting outcomes (recurrence, progression, Bacillus:Calmette-Guerin (BCG) failure) at initial diagnosis, patients may be under-treated with intravesical therapy alone or over-treated with immediate cystectomy, both with attributed adverse patient outcomes[2,3].

Multiple studies have described the importance of epigenetic modifications in tumourigenesis, most frequently apparent as inappropriate DNA methylation within gene promoter-associated CpG islands, and/or changes that lead to histone tail modification(s)[4,5].These modifications impact upon gene expression and promote tumourigenesis predominantly by silencing of tumour-suppressor and/or apoptotic pathway genes[5,6]. Such epigenetically-mediated gene silencing has been demonstrated in NMIBC and muscle-invasive bladder cancer (MIBC), and is reported to be associated with tumour recurrence, progression, invasion, and metastasis, but also early events in tumour development such as the ‘field-defect’ phenomenon[7,8]. Recent studies highlight the potential clinical utility of epigenetic biomarkers in bladder cancer, describing tumour, blood and urine DNA methylation markers of, for example, NMIBC recurrence or chemo-resistance in MIBC[9–11].

Promoter-associated CpG Island methylation of the *HOXA9*, *ISL1*, *NKX6-2*, *SPAG6*, *ZIC1* and *ZNF154* genes are frequent findings in bladder cancer. In these cases, methylation appears associated with aggressive tumour characteristics, and may independently predict disease recurrence, progression, or disease-specific mortality (DSM)[9,12,13]. However the majority of these reports examine heterogeneous cohorts, comprising predominantly low/intermediate-grade NMIBC; high-grade NMIBC has not been considered discretely for disease and/or subtype specific epigenetic modifications.

We assessed a unique cohort of patients with HG-NMIBC for inappropriate promoter-associated CpG Island methylation at initial presentation and removal of the primary tumour(s), with respect to known prospectively collected one-year outcomes of no-recurrence, recurrence, and progression (to MIBC or metastatic disease), and relative to low/intermediate-grade NMIBC. Identifying inappropriate methylation ‘at diagnosis’ of HG-NMIBC permitted a preliminary identification of novel and potentially clinically useful prognostic biomarkers.

## Materials and Methods

### Human tissue samples

The primary tumour and normal bladder tissues used were provided by the Bladder Cancer Prognosis Programme (BCPP, Nottingham Research Ethics Committee: 05/Q2404/173)[14], the University of Birmingham Human Biomaterials Resource Centre (National Research Ethics Service (North West 5): 09/H1010/75), and the University Hospitals of North Midlands NHS Trust (National Research Ethics Service (South Central–Oxford C): 12/SC/0725). All samples were obtained after informed written consent and under the approval of appropriate national ethics review boards (reference numbers stated above). All samples were confirmed histologically; repeat trans-urethral resection or cystectomy were performed, and/or intravesical therapy provided, where suggested by European Association of Urology Guidelines [15]. All primary human tissues (**Table 1**) were stored at -80°C prior to nucleic acid extraction.

**Table 1:** Patient and Tumour characteristics.

Cohort	Number	Age	Grade	Stage	Follow-up	Time to recurrence	Time to progression	Time to disease-specific mortality
		(years)	(G1/G2/G3)	(Ta/T1)	(months)	(months)	(months)	(months)
Controls	3	68 (67–74)						
Low/intermediate-grade Cohort	12	69 (47–82)	3/9/0	5/7	8 (4–11)	^A^	n/a	n/a
High-grade Cohort	36	75 (45–92)			18 (7–68)			
No-recurrence	11	75 (59–84)			59 (12–67)	n/a	n/a	^B^
Recurrence	11	84 (67–92)			31 (12–67)	5 (2–7)	^C^	16 (15–54)
Progression	14	73 (45–84)			11 (7–33)	n/a	8 (2–10)	9 (1–21)

Number of samples in each cohort, with median age, range in brackets, and median follow-up period, range in brackets, after initial tumour resection (diagnosis).

^A^One patient suffered a 2mm uni-focal recurrence at 6 months.

^B^One patient in the no-recurrence group suffered disease specific mortality at 16 months.

^C^One patient in the recurrence one-year group suffered progression at 21 months.

### DNA extraction and bisulphite modification

Genomic DNA was extracted from tumour and control tissues using a standard phenol-chloroform extraction procedure[16] and subsequently bisulphite-modified as previously described[4]. Bisulphite conversion of DNA was confirmed in all cases by successful PCR using primers specific to bisulphite-converted DNA (primer sequences provided in supporting information **S1 Table**). To increase the relative amount and stability of bisulphite-converted DNA, whole-genome amplification (WGA) was performed as previously described[4] (described further in supporting information **S1 Text).**

### Pyrosequencing of bisulphite-converted DNA

CpG island sequences were identified from the UCSC Genome Browser (http://genome.ucsc.edu/), and imported into PyroMark Assay Design 2.0 Software for primer design (Qiagen, Manchester, UK). Dependent on the frequency and density of CpG dinucleotides within the sequence of interest, designed primers encompassed 4–7 consecutive CpGs in each gene (primer sequences in **S1 Table** and details of genomic location in **S2 Table**). After PCR amplification of the target sequence, Pyrosequencing was performed using a PyroMark Q24 Pyrosequencer using PyroMark Q24 Software 2.0 and PyroMark Gold Q24 Reagents (Qiagen), as previously described [17] (**S1 Text**).

Methylation was stringently defined in tumours as comprising a mean level of methylation across the CpGs surveyed of greater than four standard deviations (4SD) above the mean in normal controls [4]. The number of tumours methylated (by this definition) for any given gene describes the *frequency* of methylation, whereas the mean percentage methylation of all CpGs surveyed describes the *mean level* of methylation for a tumour in any particular gene.

### Quantitative RT-PCR

Total RNA was extracted from control and tumour samples using a standard guanidinium thiocyanate-phenol-chloroform protocol[16], and complementary DNA synthesised as described previously[18].Thermal cycling using SYBR (III) Green was as previously described [19], with target genes normalised to glyceraldehyde-3-phosphate dehydrogenase (*GAPDH*) (**S1 Table**) as endogenous control (**S1 Text**). Relative quantification of transcript expression was performed using the 2^-ΔΔ^ cycle threshold (CT) method[20]. Reduced transcript expression in each tumour was regarded significant if lower than a 3-fold reduction relative to mean expression in control samples; the converse was true for increased transcript expression [18](**S1 Text**).

### Informatics and statistics

Microsoft Excel 2010 and STATA (v8, Stata Corporation, TX) were used to perform Fisher’s exact, Students’ t, and log-rank analyses, and sensitivity, specificity, and positive and negative predictive values of methylation with respect to clinical outcomes.

## Results

### Frequency of methylation within low/intermediate- and high-grade NMIBC

We initially determined the methylation status of the six candidate genes in the high- and low/intermediate-grade tumour cohorts by Pyrosequence analyses of bisulphite-converted DNA. Inappropriate methylation of promoter-associated CpG islands (**S2 Table**) was a frequent finding in both cohorts for the *NKX6-2*, *SPAG6*, *ZIC1* and *ZNF154* genes, relative to normal bladder (**Table 2**).

**Table 2:** Methylation frequency and mean level of methylation in low/intermediate- and high-grade tumour groups.

	Methylation Frequency			Mean Level of Methylation			
	Low/intermediate-grade	High-grade		Normal bladder control	Low/intermediate-grade	High-grade	
Gene name	Number (%)	Number (%)	*p* value	(%)	(%)	(%)	*p* value
*HOXA9*	10/12 (83.3)	20/36 (55.6)	0.167	6.1	44.0	29.3	0.057
*ISL1*	2/12 (16.7)	17/36 (47.2)	0.091	7.6	22.1	38.9	0.061
*NKX6-2*	10/12 (83.3)	32/36 (88.9)	0.631	5.4	38.9	50.2	0.126
*SPAG6*	10/12 (83.3)	32/36 (88.9)	0.631	8.2	38.5	52.1	0.097
*ZIC1*	11/12 (91.7)	31/36 (86.1)	0.999	17.3	62.2	58.6	0.545
*ZNF154*	10/12 (83.3)	32/36 (88.9)	0.631	7.6	44.1	53.1	0.318

Number and percentage of low/intermediate- and high-grade tumours that are methylated for each of the six candidate genes (left side of table) and the mean level of methylation in each cohort for each of the gene (right side of table). Methylation is defined using four standard deviations above the mean of the normal bladder controls as a cut-off, as described previously[4]. Differences in the number of tumours methylated comparing high- to low/intermediate-grade tumours were assessed using Fisher’s exact or Chi-squared tests (two-tailed), where *p*<0.05 is considered significant. Differences in mean methylation were assessed by Students t test, *p*<0.05 again considered significant.

**Table 2** shows that the methylation frequency in low/intermediate-grade tumours was lower for *ISL1* than for all other genes investigated (2/12 tumours; 16.7%), although by comparison, methylation frequency increased within HG tumours (17/36; 45.0%, *p* = 0.091). Conversely, frequency of methylation was lower in HG-NMIBCs for *HOXA9* (20/36; 55.6%) compared to low/intermediate-grade tumours (10/12; 83.3%, *p* = 0.167); (data summarised in **Table 2**).

To further investigate potential reasons for the observed differences in the frequency of methylation between the high- and low/intermediate-grade tumour cohorts for specific genes, we determined gene-specific methylation frequencies relative to the clinical characteristics of the HG tumours, encompassing one-year clinical outcomes of no-recurrence, recurrence, and progression.

**Fig 1:**
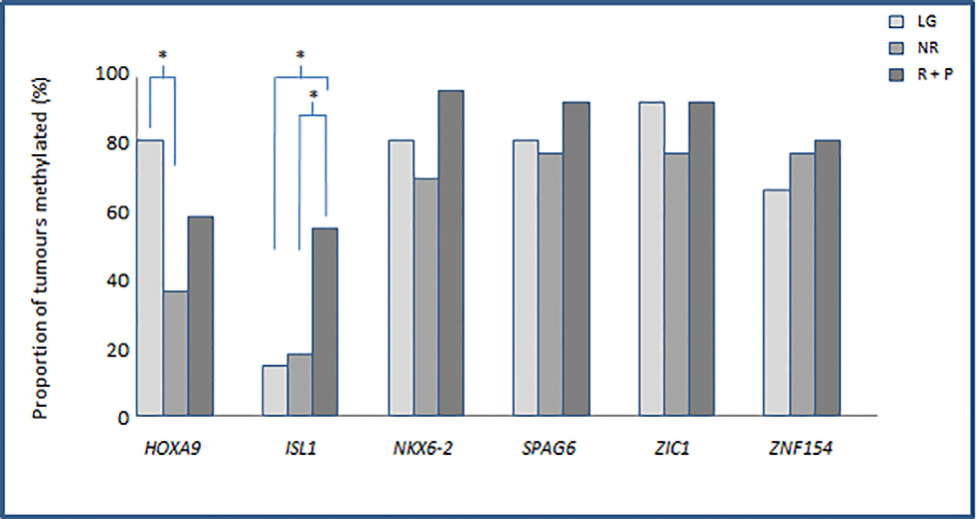
Proportion of methylated tumours in low/intermediate- and high-grade tumour cohorts. From left to right, tumour cohort methylation frequency/proportion, as determined by Pyrosequencing within *HOXA9*, *ISL1*, *SPAG6*, *NKX6-2*, *ZIC1* and *ZNF154*, for the low/Intermediate-grade cohort (LG), no-recurrence (NR) and recurrence and progression tumours (R+P) respectively. The filled bars represent the proportion of methylated tumours relative to controls in each case. Differences between the frequencies of methylation are indicated as statistically significant by ‘*’, where *p*<0.05 as determined by Fisher’s exact or Chi-squared tests (two-tailed). Methylation was defined as described in the materials and methods.

For *NKX6-2*, *SPAG6*, *ZIC1* and *ZNF154*, methylation frequencies in recurrence and progression tumours were marginally greater than their no-recurrence counterparts, and were overall broadly similar to the frequencies apparent in low/intermediate-grade tumours (**Fig 1**).

*HOXA9* demonstrated a similarly increased methylation frequency in recurrence and progression tumours relative to their no-recurrence counterparts, however, as shown in **Fig 1**, the methylation frequency in no-recurrence tumours was significantly lower compared to that observed in low/intermediate-grade tumours (*p* = 0.036). Although the trend of an increase in methylation frequency in recurrence and progression (relative to no-recurrence) tumours was apparent for all genes investigated, this was distinctly more marked for the *ISL1* gene. In this case, methylation frequency was significantly greater in recurrence and progression tumours relative to no-recurrence (15/25 (60.0%) *vs* 2/11 (18.2%), *p* = 0.031), and also to their low/intermediate-grade counterparts (15/25 (60.0%) *vs* 2/12 (16.7%), *p* = 0.017) (**Fig 1**).

### Mean methylation levels within low/intermediate- and high-grade NMIBC

**Fig 2:**
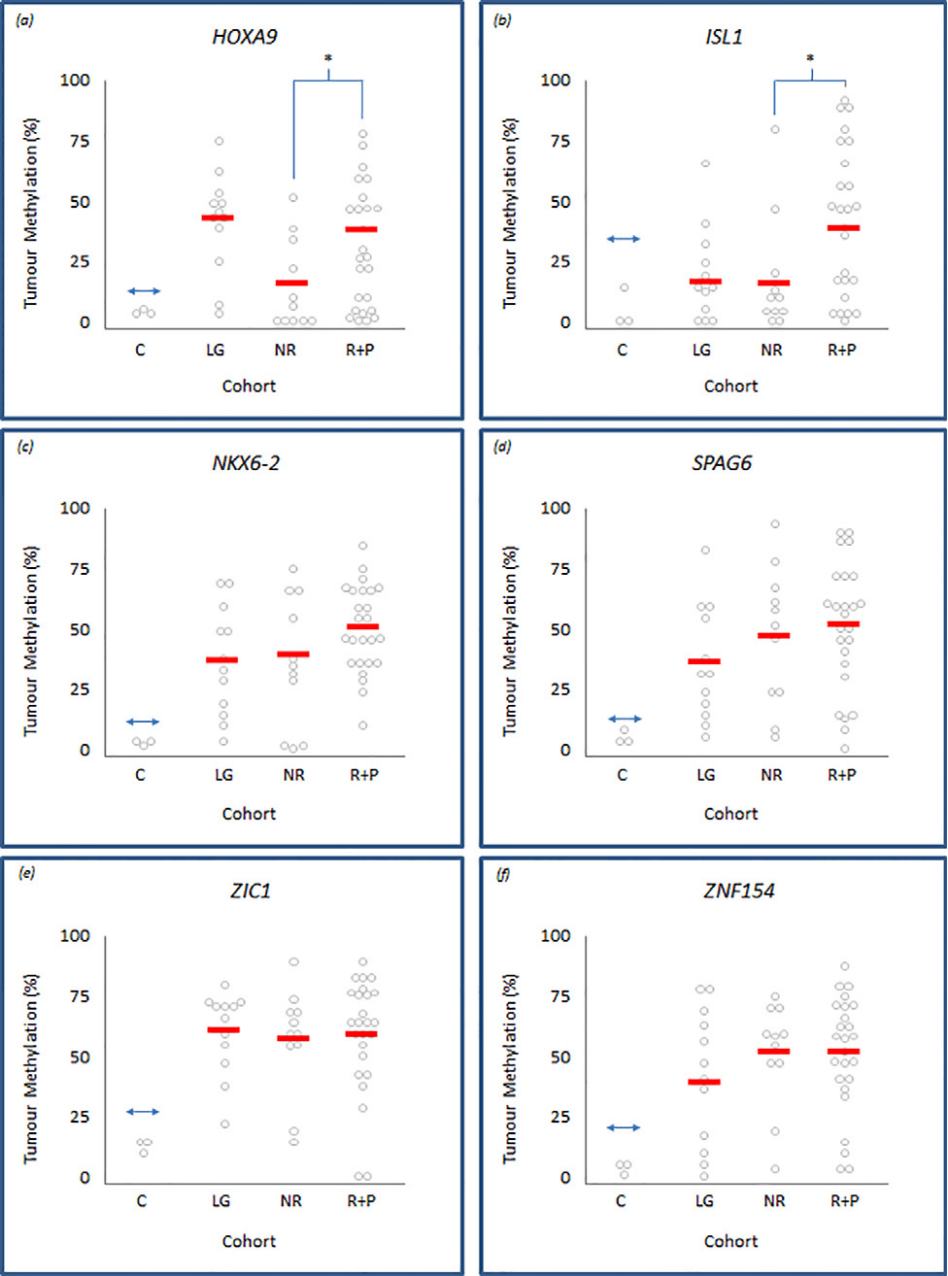
Level of methylation within low/intermediate- and high-grade tumour cohorts. Panels (a) to (f) representing *HOXA9*, *ISL1*, *NKX6-2*, *SPAG6*, *ZIC1* and *ZNF154*, respectively. Each panel displays individual tumour methylation values, as determined by Pyrosequencing, represented by grey circles within control (c), low/Intermediate-grade (LG), no-recurrence (NR) and recurrence and progression (R+P) tumour groups. The solid horizontal bars represent the overall mean methylation within each control or tumour group; differences between the means are indicated as statistically significant by ‘*’, where *p*<0.05 determined by Students-t testing. The double-headed arrow represents the cut-off point above which tumours are defined as methylated relative to normal bladder controls.

Our methylation analyses also showed considerable differences in, and between, the mean and range of methylation levels within individual tumours (**Fig 2**). Therefore, in addition to the analyses described above, we also assessed mean methylation levels within HG and low/intermediate-grade cohorts and within the HG tumour sub-types, to determine whether the level of methylation demonstrated any relationship with clinical characteristics.

The mean level of methylation was greater, albeit not significantly, in HG relative to their low/intermediate-grade counterparts for the *ISL1*, *SPAG6*, *NKX6-2*, *and ZNF154* genes (**Table 2**). This increase approached significance for *ISL1* (22.1% vs 36.5%, *p* = 0.061). Paradoxically, the mean level of methylation for *HOXA9* was lower in HG compared to their low/intermediate-grade counterparts, again approaching significance (29.3%vs 44.0%, *p* = 0.057) (**Table 2**).

Using this approach, we also determined gene-specific methylation levels within HG tumours and relative to their clinical outcomes. Mean methylation levels within *ISL1* and *HOXA9* were significantly higher in recurrence and progression tumours compared to their no-recurrence counterparts (43.3% vs. 20.9%, *p* = 0.016 and 34.5% vs. 17.6%, *p* = 0.017, respectively) (**Fig 2**). The mean methylation level increase from no-recurrence to recurrence and progression tumours was not significant for *NKX6-2*, *SPAG6*, *ZIC1* and *ZNF154*.

### Correlation of methylation within clinical outcomes of high-grade NMIBC

We next determined the correlation between gene-specific methylation with clinical outcomes within the HG tumour cohort; *HOXA9* and *ISL1* were assessed as the only genes demonstrating a significant difference in frequency or level of methylation. *HOXA9* promoter methylation demonstrated 72.7% specificity and an 84.2% positive predictive value (PPV) for tumour recurrence and/or progression within one year of initial diagnosis, whilst methylation within the *ISL1* promoter demonstrated specificity of 81.8% and PPV 87.5% for the same clinical outcomes, shown in **Table 3**. Moreover, concomitant methylation of *HOXA9* and *ISL1* at initial diagnosis predicted one-year recurrence and/or progression, with a PPV of 91.7%, whilst maintaining a specificity of 90.9%. To more rigorously assess association between these potential biomarkers and disease outcome, we employed logistical regression analysis. **Table 3** shows that when considered individually, methylation of either *HOXA9* or *ISL1* attained statistical significance with tumour recurrence and/or progression (*p* = 0.050 and *p* = 0.047 respectively). However, in combination, while the biomarkers were less significantly associated with disease outcome (*p* = 0.067), they demonstrated a stronger odds ratio of tumour recurrence and/or progression than when either was considered separately (7.86 vs 4.74 and 5.73, respectively).

**Table 3:** Methylation biomarker utility in high-grade NMIBC.

Outcome	Potential Biomarker	Odds Ratio	95% Confidence Interval	*p* value	
	*HOXA9*	4.7	1.0–22.5	0.05	
*Recurrence or Progression*	*ISL1*	5.7	2.1–32.1	0.047	
	*HOXA9 + ISL1*	7.9	0.9–71.1	0.067	
Outcome	Potential Biomarker	Sensitivity	Specificity	Positive Predictive Value	Negative Predictive Value
	*HOXA9*	64.00%	72.70%	84.20%	47.10%
*Recurrence or Progression*	*ISL1*	56.00%	81.80%	87.50%	45.00%
	*HOXA9 + ISL1*	44.00%	90.90%	91.70%	41.70%

Values for sensitivity, specificity, positive predictive value, negative predictive value, odds ratio (with 95%CI) and p-value of potential methylation biomarkers HOXA9 and ISL1 individually, and in combination, to predict high-grade NMIBC recurrence or progression at one year after initial diagnosis.

In addition to tumour behaviour we also considered promoter methylation as a predictor of disease-specific mortality: In this case, *HOXA9* promoter methylation demonstrated 57.1% specificity and a 70.6% negative predictive value (NPV) for disease-specific mortality, whilst *ISL1* methylation suggested 57.1% and 60.0% for these outcome measures.

### Methylation-associated changes in gene expression in high-grade NMIBC

**Fig 3:**
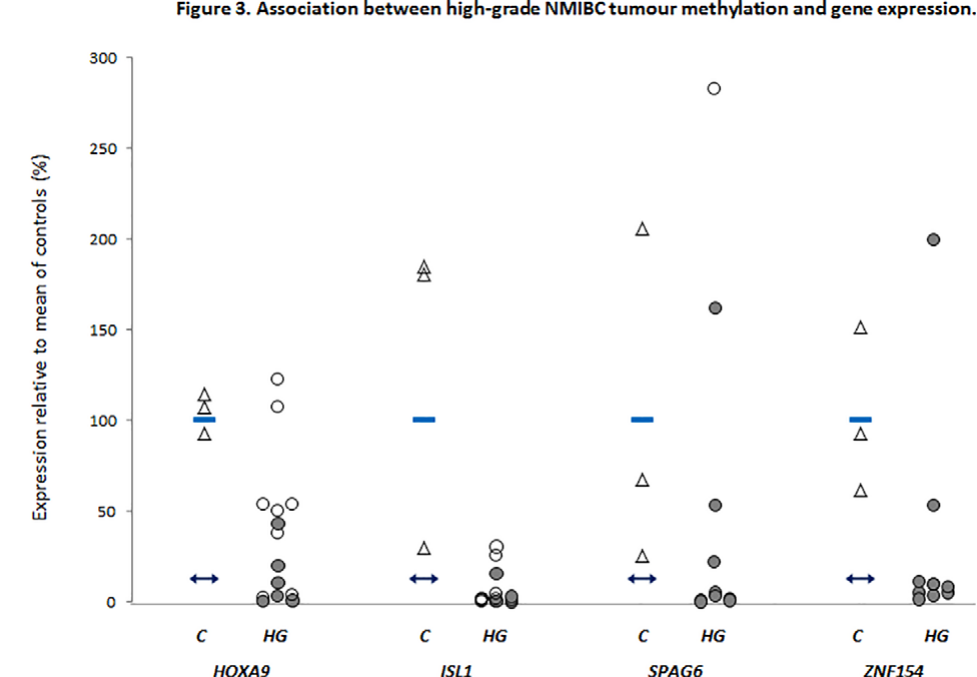
Association between high-grade NMIBC tumour methylation and gene expression. Quantitative RT-PCR analyses of *HOXA9*, *ISL1*, *SPAG6* and *ZNF154* transcript expression in individual high-grade tumours (HG). Expression is reported relative to the mean of three normal bladder controls (C, triangles), where the mean value is expressed as equal to 100%. Filled and unfilled circles denote methylated or unmethylated tumours, and represent the mean value from two independent experiments performed in triplicate. The horizontal bars within the control column represent the mean of the controls, a 3-fold reduction beneath this lies the double-headed arrow in each gene plot, representing the cut-off for significantly reduced expression in tumours relative to controls.

Quantitative RT-PCR was used to evaluate associations between methylation and gene expression in four of the six genes within a sub-set of 10–14 tumours, in comparison to controls. **Fig 3** shows that, relative to controls, 90.1% (29 of 32) methylated tumours display reduced transcript expression, and 75.0% (24 of 32) show significantly reduced expression. Conversely, 56.3% (9 of 16) unmethylated tumours displayed expression levels similar to, or in some cases higher than, that apparent in controls.

## Discussion

The epigenomic landscape of bladder cancer is an area of growing research interest[21], specifically in relation to identifying clinically viable biomarkers. Due to the current limitations in predicting the diverse clinical outcomes observed in HG-NMIBC, and the present BCG shortage, biomarkers that guide clinical decisions are of particular importance[22,23]. As described above, our analyses revealed frequent, and in some cases differential methylation, present at initial HG-NMIBC diagnosis, that appeared to correlate with tumour characteristics and clinical parameters.

The six genes selected for analyses are predominantly members of transcription factor families, primarily regulating gene-expression, enzyme-binding, and cell differentiation[24]. Their selection for analyses was on the basis of their frequent inappropriate methylation in bladder cancer [9,12,13,25]. In previous reports, *HOXA9* and *ZNF154* methylation has been associated with tumour recurrence, and *ISL1* methylation with tumour progression in predominantly low/intermediate-grade tumours[9,13].

Our initial analyses revealed similar methylation frequencies in low/intermediate- and HG-NMIBC for *NKX6-2*, *SPAG6*, *ZIC1* and *ZNF154*. In these cases, methylation frequencies were similar to those previously reported in the literature[9,12,13,25]. However, a difference in the frequency of methylation was apparent between the low/intermediate- and HG-NMIBC cohorts for *HOXA9* and *ISL1* genes: although the methylation frequency of the *HOXA9* promoter in low/intermediate-grade tumours was similar to that previously described in the literature [12,13], it was found at markedly lower frequency in HG-NMIBC. Conversely, for *ISL1*, methylation frequency in low/intermediate-grade tumours was lower than described previously by others[13]; however, the methylation frequency increase that is apparent in the recurrence and progression tumours is more consistent with these reports.

The differential methylation frequency in *HOXA9* and *ISL1* may relate to several confounders, including the method in this study employed to define tumour methylation, and the relatively few low/intermediate-grade tumours for analyses. However, the concordance between our methylation data and those reported by others for *NKX6-2*, *SPAG6*, *ZIC1* and *ZNF154*, confirms the robustness of our approach. On this basis, we reasoned that the differences in methylation frequency observed for *HOXA9* and *ISL1* may be consequent to different clinical characteristics and/or outcomes within our HG tumour cohort; a phenomenon similarly described in, for example, breast, colon and pituitary tumours[4,26,27]. We therefore performed HG-NMIBC sub-type analyses and found that the methylation profiles of recurrence and progression tumours, although similar to each other, were mostly quite distinct from their no-recurrence counterparts; on this basis we grouped recurrence and progression tumours for analyses.

The methylation frequency in recurrence and progression tumours was consistently higher across all six genes compared to their no-recurrence counterparts; these differences were most pronounced for *HOXA9* and *ISL1* and achieved statistical significance for *ISL1*. Paradoxically, in *HOXA9*, no-recurrence, recurrence and progression tumours were less frequently methylated than in their low/intermediate-grade tumour counterparts. Although the reasons for this are unclear, possible explanations may relate to the number, or heterogeneity, of tumours investigated, though we cannot discount the possibility that there are epigenetic differences in particular genes between low/intermediate- and high-grade tumours. Conversely, methylation frequency in recurrence and progression tumours in *ISL1* was markedly greater than in their low/intermediate-grade counterparts. Similar explanations to those described for *HOXA9* might account for these observations.

Through quantitative Pyrosequence analyses, we determined mean methylation across multiple CpG sites showing that, further to methylation frequency differences, there exist differences in methylation levels between tumour cohorts for particular genes. Specifically, differences in mean methylation levels between low/intermediate- and HG tumours, and also between the no-recurrence, and their recurrence and progression tumour counterparts, analogous to observations reported by others in breast and colon cancer sub-types[26,27]. For both *HOXA9* and *ISL1*, there was a significant increase in the mean level of methylation in the recurrence and progression tumours compared to their no-recurrence counterparts.

The observed differences in methylation frequency and mean methylation level between these clinically divergent subgroups, suggests that the frequency and/or mean level of methylation increases with tumour aggressiveness. Similar trends have been reported in other tumour types[26,27] and this is thought to represent accumulation of epigenetic aberrations over time, similar to the accumulation of genetic mutations and genomic instability apparent during tumour progression[28].

Since our findings support previous associations of *HOXA9* and *ISL1* methylation with tumour characteristics and behaviour[9,13], we appraised potential clinical correlates of *HOXA9* and *ISL1* methylation, including their prognostic potential. In our HG-NMIBC cohort, *HOXA9* or *ISL1* methylation at initial diagnosis reliably predicted tumour recurrence or progression within one year. In this context, concurrent methylation of both *HOXA9* and *ISL1* improved the positive predictive value to 91.7%, comparing favourably with other postulated biomarkers of recurrent/progressive disease in NMIBC [13,29]. Moreover, logistic regression appeared to confirm that these findings were not consequent to methylation in a single gene. Furthermore, and as previously described[9,13], concurrent methylation in *HOXA9* and *ISL1* was associated with DSM and reduced overall survival, however, this did not reach statistical significance. In this context, we recognise that the number of tumours available for investigation and in the absence of similar reports, that the associations we report between inappropriate methylation and HG-NMIBC disease outcome will require validation in larger, independent tumour cohorts.

Finally, to reveal potential functional relevance of methylation within high-grade disease, we assessed the relationship between methylation and gene expression; across the four genes studied, we found an association of abnormal methylation with reduced transcript expression, consistent with findings from other groups[9,13]. These findings were not significant, possibly relating to number of tumours studied, or the passenger/driver phenomenon, whereby epigenetic marks may be present but not causative of altered gene expression[30].

These findings are the first to report similarities and differences of gene-associated methylation in HG-NMIBC relative to low/intermediate-grade tumours. Furthermore, we have shown that specific methylation patterns at initial diagnosis predict one-year HG-NMIBC clinical outcomes, pointing to the exciting potential for methylation as a prognostic biomarker in this clinically unpredictable disease. Further investigations exploiting genome-wide array analyses are required to further characterise epigenetic similarities and differences between low/intermediate-and HG-NMIBC, and to reveal biomarkers that may serve as therapeutic targets or guide clinical management, such as the timing, dose and length of BCG therapy, or timing of cystectomy.

## Supporting Information

S1 Table Primer table. List of bisulphite-converted PCR primers, Pyrosequencing (sequencing) primers, and RT-qPCR primers.
(DOCX)

S2 Table CpG island information. Table listing the genomic location of the promoter-associated CpG island regions assessed for each of the six genes.
(DOCX)

S3 Table Raw data file. Excel spreadsheet containing mean values for Pyrosequencing and RT-qPCR data.
(XLSX)

S1 Text Supplemental materials and methods. Further description of primary tissue samples, and further description of DNA extraction, sodium bisulphite-conversion, Pyrosequencing, RNA extraction and RT-qPCR procedures.
(DOCX)
