# Supplementary material for: Methylation of HOXA9 and ISL1 Predicts Patient Outcome in High-Grade Non-Invasive Bladder Cancer
Source: PLoS One. 2015 Sep 2;10(9):e0137003. doi: 10.1371/journal.pone.0137003 (PMC4558003; doi:10.1371/journal.pone.0137003)
Supplement: S1 Table — List of bisulphite-converted PCR primers, Pyrosequencing (sequencing) primers, and RT-qPCR primers. (DOCX) [file pone.0137003.s001.docx]

| Gene Symbol | Forward Primer | Reverse Primer | Sequencing Primer | Amplicon Size |
| --- | --- | --- | --- | --- |
| (BSC) |  |  |  |  |
| *HOXA9* | TTTAGGGTTTTAGTGGTGGTTAT | TTCCCCCCCCATACCACCAAATTATTACA | AATTATTACATAAAATCTACAATT | 115bp |
| *ISL1* | GAGAGGGAGGTTAGAGTTAGAA | TTCCTTATCTCTTACTCAAACTTCTCTAC | GAGGTTGAAATATGATAATTAG | 284bp |
| *NKX6-2* | GGGGATTAGTTAAATAATTTATTGATGATA | TCCACTCCCAAATCTAATTCCA | AAATAATTTATTGATGATATAAAG | 369bp |
| *SPAG6* | GGGGGAGTTATGAGTTAGAGGTA | AACTAAATCCTAACCTTCTAATAT | AGGTGTTTTAATTAGTTGG | 160bp |
| *ZIC1* | GGTTTGTTAAAAGGGGATGT | ACACCCTCCCCCCCTTAAT | GTTTTATAATATTTGGGATTGA | 210bp |
| *ZNF154* | AGTGTTAAAAAAAGGGTT | CCTAAAAAACCCACCTCAAACCTA | AGATTTTGAGGGTAGAGTT | 274bp |
| (Expression) |  |  |  |  |
| *HOXA9* | GTATAGGGGCACCGCTTTTT | AATGCTGAGAATGAGAGCGG |  | 188bp |
| *ISL1* | ATGACAAAACTAATATCCAGGGG | CTGAAAAATTGACCAGTTGCTG |  | 192bp |
| *SPAG6* | TGTTGAATTGTTGGGACCAC | GCGACTAGACCCCAAAACAT |  | 247bp |
| *ZNF154* | AAGAGCCAAGTTCTCCAGCA | CACTCTGAGGACGCCAACTC |  | 208bp |
