# Supplementary material for: Methylation of HOXA9 and ISL1 Predicts Patient Outcome in High-Grade Non-Invasive Bladder Cancer
Source: PLoS One. 2015 Sep 2;10(9):e0137003. doi: 10.1371/journal.pone.0137003 (PMC4558003; doi:10.1371/journal.pone.0137003)
Supplement: S2 Table — Table listing the genomic location of the promoter-associated CpG island regions assessed for each of the six genes. (DOCX) [file pone.0137003.s002.docx]

| Gene symbol | Location of promoter region assessed |
| --- | --- |
|  |  |
| *HOXA9* | chr7:27,205,000-27,205,200 |
| *ISL1* | chr5:50,679,000-50,678,800 |
| *NKX6-2* | chr10:134,598,300-134,598,500 |
| *SPAG6* | chr10:22,634,400-22,634,600 |
| *ZIC1* | chr3:147,126,900-147,127,100 |
| *ZNF154* | chr19:58,220,400-58,220,200 |
