# Supplementary material for: Methylation of HOXA9 and ISL1 Predicts Patient Outcome in High-Grade Non-Invasive Bladder Cancer
Source: PLoS One. 2015 Sep 2;10(9):e0137003. doi: 10.1371/journal.pone.0137003 (PMC4558003; doi:10.1371/journal.pone.0137003)
Supplement: S1 Text — Further description of primary tissue samples, and further description of DNA extraction, sodium bisulphite-conversion, Pyrosequencing, RNA extraction and RT-qPCR procedures. (DOCX) [file pone.0137003.s004.docx]

**S1 Text. Supplemental Materials and Methods**

Human tissue samples

The primary HG-NMIBC and bladder control samples were confirmed histologically as normal bladder urothelium (control), or as G3 pT1 TCC (HG-NMIBC). HG-NMIBC samples were excluded if carcinoma-in-situ (CIS) was also present in any of the resected specimens, satellite lesions or biopsies. Primary low/intermediate-grade NMIBC tumours were confirmed histologically as before.

DNA extraction and bisulphite modification

Genomic DNA was extracted from tumour and control tissues using a standard phenol-chloroform extraction procedure ([16](#_ENREF_16)), dissolved in molecular biology grade water (Sigma Aldrich, Dorset, UK), then assessed and quantified by spectrophotometry on a NanoDrop 2000 (Thermo Scientific, Loughborough, UK). Sodium bisulphite modification of 500ng genomic DNA was performed using EZ DNA Methylation Gold Kit™ (Zymo Research, Cambridge, UK), using the manufacturer’s protocol as described previously ([18](#_ENREF_18)). Bisulphite-conversion of DNA was confirmed in all cases by successful PCR using primers designed for bisulphite-converted DNA in a region of the *ZNF154* gene (primer sequences in supporting information **S1 Table**). To increase the relative amount and stability of BSC DNA, whole genome amplification (WGA) was performed on 4 µL converted DNA, by a primer extension pre-amplification (PEP) method using Taq DNA Polymerase (Promega BioSciences, CA, USA), as described previously by us ([4](#_ENREF_4)).

Pyrosequencing™ of bisulphite-converted DNA

For six genes reported in literature as harbouring promoter-associated CGI methylation in bladder cancer, their associated CpG island sequences were identified from the UCSC Genome Browser (http://genome.ucsc.edu/), and imported into PyroMark Assay Design 2.0 Software for primer design of sodium bisulphite-converted DNA (Qiagen, Manchester, UK). Dependent on the frequency and density of CpG dinucleotides within the sequence of interest, primers were designed to interrogate between 4 and 7 consecutive CpGs in each gene (supporting information **S2 Table**). For each gene, 2 μL of WGA bisulphite-converted DNA were used as template in a PCR as previously described ([4](#_ENREF_4)). The capture of biotinylated amplicons was performed to the manufacturers’ instructions, using a Pyromark Q96 ‘Vacuum Prep’ workstation. Pyrosequencing™ (PSQ) was performed using a PyroMark Q24 Pyrosequencer as previously described ([17](#_ENREF_17)) (Qiagen). *In-vitro* methylated DNA standards or sample repeats were included as internal controls and to allow comparison between runs.

Quantitative RT-PCR

Total RNA was extracted from control and tumour samples using a standard guanidinium thiocyanate-phenol-chloroform protocol as described previously ([16](#_ENREF_16)). RNA pellets were dissolved in molecular grade water then assessed and quantified by spectrophotometry on a NanoDrop 2000. Complementary DNA (cDNA) was synthesised using 200U M-MLV reverse transcriptase (Promega), using the manufacturers protocol and as described by us previously([17](#_ENREF_17)).

Thermal cycling step conditions were as previously described ([18](#_ENREF_18)), namely an initial denaturation phase followed by two-step denaturation and annealing for 40 cycles, using Brilliant III SYBR Green QPCR Master mix (Agilent Technologies, California, USA).

The target genes were normalised to an endogenous control gene (*GAPDH*), and relative quantification of transcript expression was performed using the 2^-∆∆^ cycle threshold (CT) method ([20](#_ENREF_20)), where -∆∆CT = CT^(gene of interest of tumour -^ *^GAPDH^* ^of tumour)^ - CT^(gene of interest of control - GAPDH of control)^. Loss or reduced transcript expression in each tumour was regarded as significant if lower than four standard deviations (4SD) below the mean expression of the control samples, as previously described ([4](#_ENREF_4)). The sequences for primers used for quantitative RT-PCR are shown in supporting information **S1 Table.**
